# Targeted dream incubation at sleep onset increases post-sleep creative performance

**DOI:** 10.1038/s41598-023-31361-w

**Published:** 2023-05-15

**Authors:** Adam Haar Horowitz, Kathleen Esfahany, Tomás Vega Gálvez, Pattie Maes, Robert Stickgold

**Affiliations:** 1 MIT Media Lab, Massachusetts Institute of Technology, Cambridge, MA 02139 USA; 2 Center for Sleep and Cognition and Department of Psychiatry, Beth Israel Deaconess Medical Center, Boston, MA 02215 USA; 3 Department of Psychiatry, Harvard Medical School, Boston, MA 02115 USA; 4 Department of Brain and Cognitive Sciences, Massachusetts Institute of Technology, Cambridge, MA 02139 USA

**Keywords:** Circadian rhythms and sleep, Human behaviour

## Abstract

The link between dreams and creativity has been a topic of intense speculation. Recent scientific findings suggest that sleep onset (known as N1) may be an ideal brain state for creative ideation. However, the specific link between N1 dream content and creativity has remained unclear. To investigate the contribution of N1 dream content to creative performance, we administered targeted dream incubation (a protocol that presents auditory cues at sleep onset to introduce specific themes into dreams) and collected dream reports to measure incorporation of the selected theme into dream content. We then assessed creative performance using a set of three theme-related creativity tasks. Our findings show enhanced creative performance and greater semantic distance in task responses following a period of N1 sleep as compared to wake, corroborating recent work identifying N1 as a creative sweet spot and offering novel evidence for N1 enabling a cognitive state with greater associative divergence. We further demonstrate that successful N1 dream incubation enhances creative performance more than N1 sleep alone. To our knowledge, this is the first controlled experiment investigating a direct role of incubating dream content in the enhancement of creative performance.

## Introduction

Creative thinking is essential to our functioning, yet often elusive. One of the most well-studied and longstanding theories of creativity is the associative theory, which proposes that creative solutions can result from identifying remote associations between existing concepts stored in memory^1,2^. Under this framework, the ideal cognitive state for creative idea generation is one which promotes a broadened representational search space to encounter novel associations, while still maintaining enough control to evaluate and identify those best suited for the task at hand^2,3^.

The link between dreams and creativity has been a topic of intense speculation for millennia. Anecdotal reports of scientific and artistic discoveries made while dreaming by the likes of Thomas Edison and Salvador Dalí emphasized dreams occurring in the transition from wakefulness into sleep, a period also known as hypnagogia or NREM1 (N1)^4^. The N1 sleep stage is characterized as a period containing spontaneous, vivid dreams which often incorporate awake experiences occurring shortly before sleep onset^5–7^. The basic technique used by Edison and Dalí for capturing hypnagogic insights consisted of dozing off with a heavy object in hand. Once muscle tone lessened at sleep onset, the object would drop, waking the sleeper, who then recalled and recorded potential insights made in their hypnagogic dreaming.

In addition to these anecdotal reports of insights made while dreaming, scientific studies largely focused on REM sleep have suggested that sleep may present an optimal brain state for creative ideation. Periods of sleep are known to foster insight over and above time matched periods of wake^8–11^. Neuroimaging data suggest that the functional connectivity of higher-order associative areas of the brain during REM sleep favors associations between distant memories^12^. A recent study on the sleep onset stage of N1 has suggested that N1 is a creative sweet spot, finding that spending as little as 15 s in N1 sleep tripled the chance of participants subsequently having a moment of creative insight on a previously studied mathematical task as compared to participants who remained awake^13^. Importantly, if participants fell past N1 sleep into N2, this creative benefit was lost. These results linking N1 sleep with enhanced creativity make sense within the associative framework of creativity. The N1 sleep state is characterized by less constrained cognitive control than wake, while preserving enough control for the recall of task-relevant ideation, facilitating the exploration and capture of remotely associated concepts^13^.

Although research has shown that sleep may promote creative ideation, the scientific literature linking dreaming and creativity remains sparse. Few experiments have collected relevant data on the phenomenological dream content that may contribute to creativity, instead mostly correlating sleep physiology with waking creative traits. For example, one study presented participants with a creativity task accompanied by a specific odor^14^. The study found higher creative performance on the task following the presentation of a task-related odor during overnight sleep compared to different-odor and no-odor control conditions. The task-relevant odor presumably reactivated memories of the creativity task during sleep, prompting creative ideation. However, this study did not report any data about the dreams which accompanied, and potentially drove, this creative processing^14^. Even in studies that have collected phenomenological reports, many have limited analysis of dream content. For example, in the study of sleep onset as a creative sweet spot described above, 36% of dream reports were excluded from analysis due to not fitting the study criteria for hypnagogic reports, which required reports to be “fleeting, involuntary, spontaneous, perceptual, and bizarre” in content. No correlation was found between hypnagogic dreams and post-sleep performance^13^. Still, other studies which have collected and analyzed relevant data on sleep phenomenology and creativity have shown that dream recall frequency and dream complexity are correlated with higher creativity^15–17^.

Several studies investigating the link between dreaming and various other task domains have collected relevant phenomenological data and have found dream content related to a pre-sleep task correlates with enhanced post-sleep task performance. Dream incorporation of words from a foreign language is correlated with improved performance on language learning tasks^18^. Dream incorporation of words from a story is correlated with improved performance on story recall tasks^19,20^. Reporting a dream about an exam from the pre-exam night is associated with better performance on the exam, and the frequency of dreams concerning the exam during a school term correlates with exam performance^21^. In N1 dreams, dream incorporation of novel learning experiences is thought to reflect the processing of the newly learned material^22,23^. Improved coordination on a tennis video game task is correlated with gameplay incorporation into hypnagogic dreams, but not incorporation into daydreams^24^. Participants trained on a 3D virtual maze task who refer to the maze task in their hypnagogic dream reports improve ten-fold compared to participants who give no task-related dream reports. Moreover, thinking about the maze while awake was not associated with any significant performance benefit^25,26^. In all of these studies, task-relevant dream experiences reflect the reactivation of memories during sleep, and the phenomenological recall of experiences of this reactivation (i.e., dreams) correlates with a subsequent enhancement of memory performance.

To make a causal claim about the effect of dream content on post-sleep performance, a controlled experiment must be conducted in which dream content is independently varied across randomly assigned groups^27^. As such, previous studies aiming to link dream content with waking performance faced a key methodological challenge: dream content is difficult to control^28^. Many studies have followed the approach of presenting a task pre-sleep to all participants, identifying participants who spontaneously had task-related dreams afterwards, and then correlating dream content with post-sleep performance. A variation on this protocol involves the use of a sensory-level intervention during sleep called targeted memory reactivation (TMR)^29^. TMR relies on continued sensory processing of sounds, scents, and somatosensory input during sleep^30^. In TMR, a sensory cue that was previously linked to a task pre-sleep is re-presented during sleep to drive specific reactivation of task-related memories associated with the cue, and it has been shown to improve post-sleep performance on tests of declarative memory, skill learning, and spatial navigation^31–33^.

Key limitations of this approach constrain these studies’ claims about dream function and post-sleep performance to be correlative rather than causal. The first limitation is the use of the task itself pre-sleep^13,25,26^. Since previous studies have already demonstrated that intervening periods of sleep improve memory consolidation on tasks presented pre-sleep (ignoring any intervening dream content), presenting the task pre-sleep makes it unclear whether task performance independently causes sleep-dependent performance improvement and related dreams, or if dreaming itself mediates this performance improvement. Furthermore, this approach is limited by a lack of manipulation of dream content as an independent variable across randomly assigned groups. For example, in one study, participants asked to think of or “incubate” a problem of their choosing in their dreams frequently self-reported dreaming of a useful solution, but the study had no control group of non-incubated dreaming, further limiting interpretation of the specific contribution of dream content rather than other, unconscious processing^34,35^.

A relatively novel protocol called targeted dream incubation (TDI) addresses these core methodological challenges by incubating specific themes in dreams without any pre-sleep task^36,37^. Similar to TMR, TDI relies on the continued sensory processing of sound during the sleep onset period as an avenue for introducing specific themes into dream content^30^. However, unlike TMR, TDI does not rely on the pre-sleep presentation of a task, allowing for tasks to be presented exclusively post-sleep, thus eliminating the possible explanation of pre-sleep task performance independently affecting dream content and post-sleep task performance. Additionally, TDI facilitates the manipulation of dream content as an independent variable across randomly assigned groups, allowing for controlled studies of dream content.

To investigate the role of incubating N1 dream content on post-sleep creative performance, we administered a TDI protocol during N1 and presented creativity tasks post-sleep. We used TDI to incubate a specific theme (a “tree”) and assessed creative performance using three tasks related to this theme. We first compared creative performance following a period of N1 sleep or wake to corroborate recent findings identifying N1 as a creative sweet spot. We then measured semantic distance in task responses to test the hypothesis that N1 enables a cognitive state promoting the exploration of more distantly associated concepts. To zero in on the specific potential contribution of dream content to creativity, we then analyzed creative performance as a function of the successful incubation of the “tree” theme in dream content. This study thus offers the first controlled experimental design to study the effects of incubating N1 dream content on creative performance.

## Methods

All research procedures were approved by the MIT Institutional Review Board and the MIT Committee on the Use of Humans as Experimental Subjects and were performed in accordance with relevant guidelines and regulations, including the standards set forth in the Declaration of Helsinki. Informed consent was obtained from all participants.

### Participants

We recruited 50 healthy participants (mean age = 26.7 ± S.D. 7.9 years, 24 females) to participate in a daytime napping study using an email advertisement sent to a university listserv containing MIT-affiliated students and researchers. Participants were screened for exclusion criteria of any self-reported history of sleep or psychiatric disorders. Participants arrived at the laboratory in the afternoon between the hours of 12:00 pm and 4:00 pm, optimizing for the postprandial increase in sleepiness. Participants were informed the study investigated the relationship between rest and cognitive flexibility and that they would engage in a nap or active rest. They were offered a sleep mask as compensation for participation in the study. Participants were instructed not to consume stimulants on the day of the experiment. All participants signed an informed consent form. After signing the consent form, participants filled out questionnaires on their demographic information and typical sleep quality.

### Design

**Figure 1:**
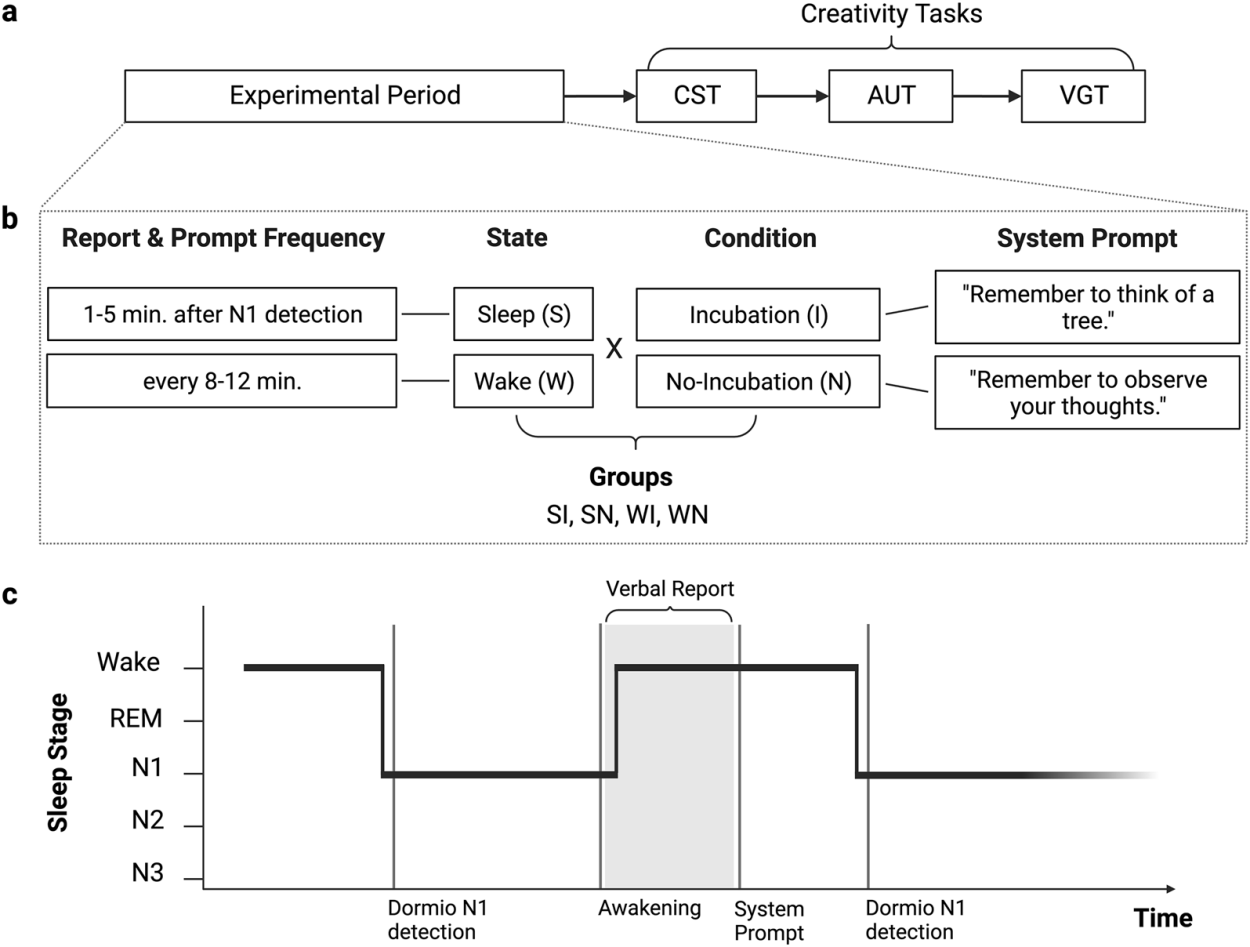
Experimental protocol. (**a**) Flowchart indicating the order of events in the experimental protocol. The period of sleep or wake was 45 min in length. CST: Creative Storytelling Task; AUT: Alternative Uses Task; VGT: Verb Generation Task. (**b**) Verbal report and system prompt parameters used in the experimental period for each independent variable level. (**c**) Hypnogram schematic displaying sleep staging for sleep participants. Participants repeatedly transitioned between wakefulness and N1 sleep throughout the experimental period. After the detection of N1 and a variable period of 1–5 min, participants were awakened and asked for a verbal report. After their verbal report, the Dormio system delivered a prompt and the participant was left undisturbed to return to sleep.

The experiment used a 2 × 2 between-subjects design in which the independent variables were state (Sleep or Wake) and condition (Incubation or No-Incubation). Using these states and conditions, we generated four groups to which participants were randomly assigned: Sleep Incubation (SI), Sleep No-Incubation (SN), Wake Incubation (WI), and Wake No-Incubation (WN) (Fig. 1b). Participants engaged in a 45-min experimental period and then completed three creativity assessments (Fig. 1a). One participant assigned to a sleep group was unable to sleep and was eliminated from analysis, leaving a total of 49 participants.

### The Dormio device

Regardless of their assigned group, all participants wore the Dormio device during the 45-min experimental period. The Dormio system consists of a hand-worn sleep tracker and associated app installed on a laptop or smartphone^36,37^ (Fig. S1a–c). The Dormio system was used to automatically track participants’ sleep onset, communicate auditory cues to participants, and record participants’ verbal reports (Fig. 1)^4,36–39^. The Dormio device uses physiological sensors to automatically detect sleep onset, i.e., the transition from wake to N1. During the development of the Dormio device, concurrent polysomnographic data^38^ (Fig. S1) identified the following changes as markers of sleep onset for use in the Dormio system: heart rate changes of > 5 BPM, electrodermal activity sensor changes > 4 μSiemen, or flexor muscle sensor changes > 8 KΩ. For participants in the Sleep groups, surpassing one of these thresholds triggered a variable timer of 1–5 min, after which the Dormio system delivered an auditory prompt to wake the participant and collected a verbal report (Fig. 1). Verbal reports were not subject to a time limit. Additional pilot data indicated that this protocol not only corresponded well with entry into N1 sleep but also effectively limited participants’ entry into N2 sleep^36,37^. Note that due to the lack of polysomnographic confirmation of sleep staging within the study (such as with EEG), the term “N1 sleep” throughout our study refers to the period defined as 1–5 min after Dormio-detected sleep-onset N1.

### Targeted dream incubation protocol

Targeted dream incubation (TDI) is a protocol designed for the induction of specific content into sleep-onset dreams, allowing for controlled studies using dream reports as an independent variable^36,37^. TDI requires sensors to track sleep onset and a method to deliver and record audio. The Dormio device enacts the TDI protocol automatically by delivering audio cues to suggest a dream theme to participants during each of a series of awakenings, creating a serial dream incubation paradigm^36^. In addition, Dormio enables an automated version of a manually run serial awakenings paradigm previously used to collect hypnagogic dream reports^40,41^. Using Dormio, each awakening from a dream incubation period is accompanied by an auditory prompt that requests a dream report, which the Dormio system automatically records (see Supplementary Table S1 for sample reports)^36^.

### Experimental protocol

We used Dormio to enact a serial auditory incubation of the theme “tree.” The data collected on the efficacy of the Dormio dream incubation in these participants have been previously reported^36^, while all data related to post-sleep creativity task performance are reported for the first time here. All participants engaged in a 45-min experimental period in which protocols differed based on their assigned state and condition. The precise wording of both the pre-experiment instructions (read by experimenters to participants) and the Dormio-delivered pre-recorded prompts (for theme incubation and requesting verbal reports) for each group can be found in the Supplementary Materials and are summarized below.

- *SI*: For the Sleep Incubation group, a variable timer initiated wakeups 1 to 5 min after Dormio detected entry into N1 sleep. During each wakeup, a computer with pre-recorded audio prompts requested a verbal dream report from the participant, which was recorded on the computer. Another pre-recorded voice prompt instructed participants to “remember to think of a tree” and go back to sleep.
- *SN*: The Sleep No-Incubation group followed a similar protocol of awakenings and dream reports, except that they were instructed to observe their thoughts (“remember to observe your thoughts”) rather than think of a tree.
- *WI*: In the Wake Incubation group, participants were left to mind-wander for periods of 7 min (mirroring the average time needed for sleep onset in the sleep groups) plus a variable period of 1 to 5 min. Following each period, the computer requested and recorded a verbal report from the participant about their thoughts and then instructed them to “continue thinking about a tree.”
- *WN*: The Wake No-Incubation group followed a similar protocol of mind-wandering and verbal reports, except that they were instructed to observe their thoughts rather than to think of a tree.

Throughout the 45-min experimental period, experimenters remained in the room with the participant. Experimenters were out of sight, as participants assigned to sleep wore an eye mask and participants assigned to stay awake were instructed to have their eyes closed. Following the experimental period and completion of the creativity tasks, we also collected written reports from participants about their mentation during the experimental period (see Supplementary Table S2 for sample post-experiment written reports).

### Assessing theme incorporation in verbal reports

Throughout this study, “incubation” refers to the process of providing stimuli to direct dreaming or awake mentation towards a specific theme, as in “incubating” a specific dream topic. With each incubation group, participants experienced different degrees of successful incubation, reflected in differences in how many times their verbal reports referenced the “tree” theme. We quantified the degree of theme incorporation for each participant as the average number of direct references to the incubated theme (“tree”) made in each verbal report. A direct reference to “tree” is defined as an unambiguous mention of “tree” or part of a tree (including leaf, branch, root, or forest), adapting methods from Wamsley et al.^40^. For example, there was 1 direct reference in the report “*trees splitting into infinite pieces”* and there were 3 direct references in the report *“thinking about how leaves are the eyes, roots are like lips, and the different senses trees must have”* (counting the words “leaves,” “roots,” and “trees” as one reference each).

### Creativity tasks

Due to its highly context-dependent nature, constructing a single definition or assessment of creativity has proven to be a major challenge for the sciences^42^. In many cases, studies have used just one assessment tool, leading to results constrained in their real-world relevance. In the present study, all participants completed a set of three widely validated creativity assessments, each shown to index a different aspect of creative performance, in order to facilitate a multifaceted measurement of creativity with broad real-world relevance. Immediately following the 45-min experimental period, participants completed the creativity assessments in the following order: the Creative Storytelling Task^43,44^, the Alternative Uses Task^45,46^, and the Verb Generation Task^47^.

The Creative Storytelling Task (CST) has been used for decades to assess creative effort, semantic divergence, and the ability to make new combinations of mental elements that form the basis of a meaningful product. Importantly, the CST assesses abilities with real-world relevance for individuals engaging in creative writing in their professional or personal lives. The CST has been used to assess the neural correlates of creativity and has been shown to engage brain areas core to creativity^43,44^. In this task, all participants were instructed to write a creative story including the word “tree.” Participants wrote responses by hand. Participants were instructed to use their imagination and to be creative. They were told writing was necessary but drawing was additionally allowed. No time limit was given, but every 5 min that passed was announced so participants could have a sense of time, and total writing time was recorded. Writing times ranged from 2 to 19 min (mean time = 8.9 ± S.D. 3.9 min).

The Alternative Uses Task (AUT) has been widely validated as a measure of creativity that specifically indexes divergent thinking abilities, namely the ability to broaden representational search space and produce wide-ranging responses to queries. Furthermore, task performance is correlated with and predictive of real-world creative achievements^46,48^. For this task, participants were prompted to “list all the creative, alternative uses you can think of for a tree” and given three minutes to provide handwritten responses. Participants were explicitly told to be creative, as noted in the prompt. Explicit instructions to be creative have been consistently shown to influence creativity across a wide variety of tasks, including divergent thinking tasks^49^.

The Verb Generation Task (VGT), a classic cognitive neuroscience measure of language production and semantic processing abilities, has been used to quantify the contribution of semantic divergence to creativity^49^. Performance on the task can be automatically computationally assessed, and these objective assessments have been shown to correlate well with subjective assessments by raters^50^. For this task, a list of 31 nouns was presented and the participants were instructed to respond in writing with the first verb that came to mind for each noun. No time limit was given, but participants answered each noun prompt in under 10 s. Participants were instructed explicitly to generate creative verb associations, adapting methods from Heinen et al.^47^. The following nouns were presented: tree, forest, leaf, roots, branch, stick, plant, seed, apple, flower, grass, sunshine, shadow, wind, water, river, dirt, shovel, hole, squirrel, bird, frog, swing, rope, nest, chair, book, mountain, tea, pot, syrup.

### Creative performance ratings

Human raters evaluated the degree of creativity in participants’ task responses.

CST responses were evaluated by three trained, state and condition-blind raters. Raters were provided stories in two formats: typed text and scans of the page containing participants’ original handwritten words and accompanying imagery. Raters assessed the “overall creativity” of participants’ stories, adapted from Prabhakaran and defined as: the extent to which the participant told a unique story that "came alive”; the story did not need to be cohesive or have a plot^49^. Raters were instructed to score CST responses on a Likert scale from 1 (low creativity) to 7 (high creativity). In line with the Consensual Assessment Technique, after ratings were completed, raters discussed differences in ratings until a consensus (defined as ratings with difference of ≤ 2) was reached^51,52^. Then, a mean across the three raters’ scores was taken for each participant. While “overall creativity” was our measure of interest, in order to fully replicate the scoring procedures from Prabhakaran, raters also assessed the following four factors for each story: descriptiveness (the extent to which the participant added additional details), semantic flexibility (the manner and number of unique ways in which the participant used the word “tree”), humor (the extent to which the participant incorporated clever, witty, and/or amusing elements into the story), and emotiveness (the extent to which the participant used words that convey emotion and shifts of emotion). These additional factors are outside of the scope of the present study and are excluded from our analyses.

AUT responses were evaluated by the same three raters as for the CST rating protocol. Raters scored the participant’s entire set of alternative uses as a group. For this task, creativity was defined for raters as a combination of originality (uniqueness of ideas), flexibility (variety of ideas), and fluency (number of ideas), with definitions for each of these constructs taken directly from the Torrance Tests of Creative Thinking^53^. Raters evaluated the creativity of a participant’s list of alternative uses by assigning a value of 1 (low creativity) to 5 (high creativity). After ratings were assigned, the Consensual Assessment Technique^51^ was again used to address any discrepancy of ≥ 3 between any of the three raters. Then, a mean across the three raters’ scores was taken for each participant.

VGT responses were evaluated by 39 “Master Workers” recruited on the Amazon Mechanical Turk platform. We first extracted the set of unique pairings of noun prompts and verb responses across all 49 participants’ VGT responses, resulting in a total of 1219 noun–verb pairings. We then obtained scores from 5 raters for each noun–verb pairing (resulting in a total of 6,095 data points with an average of 156.3 ratings provided per rater). Raters were presented with one noun–verb pair and asked to assign a score of 1 (“Not creative at all”), 2 (“A little creative”), 3 (“Somewhat creative”), 4 (“Creative”), or 5 (“Very Creative”) to the verb with respect to the noun. We scored each noun–verb pairing by taking a mean across the 5 MTurkers’ ratings. Finally, a mean was taken for each participant across their 31 noun–verb pairings.

### Creativity Index

The three creativity tasks had different structures and indexed different aspects of creative performance. Partial Pearson correlations were calculated between task creativity ratings (with state and condition as covariates) and confirmed only moderate levels of correlations between creative performance on each task. To combine performance across all three tasks into a single multifaceted measure of creativity with broad real-world relevance, we constructed a composite measure called the “Creativity Index”. We compute the Creativity Index by first computing z-scores of creativity ratings using population means and standard deviations for each task, then averaging each participant’s z-scored creativity ratings across the three tasks^54,55^.

### Creative self efficacy

Creative self-efficacy (CSE) is defined as “the belief that one has the ability to produce creative outcomes”^56^. CSE has been shown to correlate with performance on creativity tasks^57,58^. Participants completed a creative self-efficacy survey with the question “Do you consider yourself a creative thinker?” pre-experiment (before the period of sleep or wake). Participants responded on a scale of 1 (“Not so much”) to 10 (“Yes”). The results of this survey, presented in Table 1, were used as a proxy for assessing baseline creative abilities pre-experiment, since the creativity tasks could not be presented pre-experiment (in order to avoid the tasks acting as incubation stimuli). An ART ANOVA found no significant effect for state (p = 0.29), condition (p = 0.23), and no significant interaction effect (p = 0.99). An ART multifactor contrast test with Holm-Bonferroni correction found no significant differences between groups (adjusted p = 1.00 for all contrasts).

**Table 1:** Participant verbal report and task response metrics.

	SI	SN	WI	WN
N	13	12	12	12
Female	7 (54%)	6 (50%)	5 (42%)	8 (67%)
Age	24.38 ± 4.22 (1.22)	28.50 ± 12.9 (3.89)	26.83 ± 4.9 (1.48)	27.33 ± 5.27 (1.59)
Baseline Creative Self-Efficacy	7.54 ± 1.34 (0.39)	8.08 ± 1.11 (0.34)	7.18 ± 2.08 (0.66)	7.55 ± 1.16 (0.37)
**Verbal Reports**				
# Reports	5.15 ± 1.41 (0.41)	5.75 ± 2.13 (0.64)	4.66 ± 0.62 (0.19)	4.00 ± 0.37 (0.11)
% Reports w/ “tree” reference	70.29 ± 33.66 (9.12)	1.39 ± 4.61 (1.39)	52.36 ± 34.92 (10.53)	0.0 ± 0.0 (0.0)
# “Tree” references per report	1.25 ± 0.79 (0.228)	0.01 ± 0.05 (0.014)	0.9 ± 1.03 (0.309)	0.0 ± 0.0 (0.0)
Report length (# words)	14.00 ± 9.86 (2.85)	8.71 ± 4.17 (1.26)	22.78 ± 23.67 (7.14)	18.58 ± 7.75 (2.34)
**Creativity Ratings**				
Creativity Index	0.79 ± 0.50 (0.15)	− 0.07 ± 0.70 (0.21)	− 0.32 ± 0.71 (0.21)	− 0.47 ± 0.77 (0.23)
CST	5.74 ± 0.99 (0.29)	4.78 ± 1.32 (0.40)	4.11 ± 1.27 (0.38)	4.17 ± 1.48 (0.45)
AUT	4.21 ± 0.72 (0.21)	3.25 ± 0.88 (0.27)	2.69 ± 0.99 (0.30)	2.58 ± 1.13 (0.34)
VGT	3.06 ± 0.31 (0.09)	2.65 ± 0.40 (0.12)	2.73 ± 0.28 (0.08)	2.58 ± 0.31 (0.10)
**Semantic Distance**				
Semantic Distance Index	0.37 ± 0.45 (0.13)	0.15 ± 0.65 (0.20)	− 0.22 ± 0.62 (0.19)	− 0.33 ± 0.73 (0.22)
CST	0.68 ± 0.04 (0.01)	0.68 ± 0.03 (0.01)	0.66 ± 0.03 (0.01)	0.67 ± 0.04 (0.01)
AUT	0.66 ± 0.04 (0.01)	0.64 ± 0.05 (0.02)	0.62 ± 0.05 (0.02)	0.59 ± 0.07 (0.02)
VGT	0.74 ± 0.05 (0.02)	0.72 ± 0.07 (0.02)	0.70 ± 0.07 (0.02)	0.69 ± 0.05 (0.02)
**Verbal report concept incorporation in task responses (# participants)**				
CST	8	1	3	1
AUT	4	0	0	1
VGT	0	0	0	0

(Top) *N*: Number of participants in each group, *Female*: Number and percent of female participants in each group, *Age*: Mean ± Std. Dev (S.E.M.). (Middle) *Creative Self-Efficacy, Verbal Reports, Creativity Ratings, and Semantic Distance*: Mean ± Std. Dev. (S.E.M.) for creative self-efficacy, verbal reports, creativity task response ratings (including the composite Creativity Index and the creativity ratings of individual task responses), and semantic distance metrics (including the Semantic Distance Index and measurement of semantic distance in individual task responses). (Bottom) *Verbal Report Concept Incorporation in Task Responses*: Number of participants for each task where content from the participant’s verbal reports was incorporated into their task response.

### Semantic distance

We computationally evaluated the semantic distance in participant responses using a pre-trained semantic model called GloVe (Global Vectors for Word Representation)^59^. GloVe consists of a 300-dimensional word embedding space created by an unsupervised learning algorithm trained on 42 billion word tokens and their co-occurrence statistics. To evaluate the semantic distance between two words, we quantify the cosine distance between their GloVe embeddings (300-dimensional vectors). GloVe has shown robust associations with human judgments of word relatedness, comparable to other semantic models^59^.

**Figure 2:**
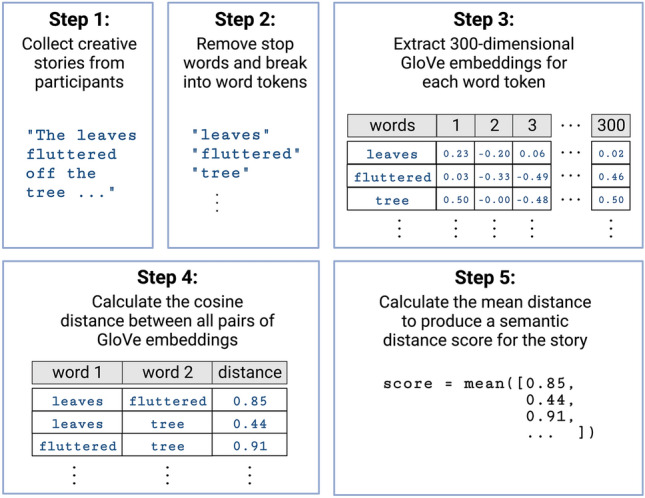
Divergent Semantic Integration with GloVe. DSI^60^ with GloVe was used to measure the semantic distance in the Creative Storytelling Task. Stories were first stripped of punctuation and stop words and then broken into word tokens. The cosine distance was computed between all pairs of GloVe embeddings. The mean of the pairwise distances was taken to produce a semantic distance score for each story. See^60^ for more details.

For the CST, we used the Divergent Semantic Integration (DSI) tool to score semantic distance^60^. Using DSI, stories were first stripped of punctuation and stop words and then tokenized. The GloVe embedding of each word token was extracted and the cosine distance was computed between all pairs of word embeddings (Fig. 2). A single semantic distance score for each story was calculated by taking the mean of this set of pairwise cosine distances.

For the AUT, semantic distance for each alternative use was evaluated by taking the GloVe cosine distance between the prompt word “tree” and the alternative use. For multiple-word use cases (such as “clothes hanger”), the semantic distance was calculated by taking the mean of the semantic distances between each component word in the response and the word “tree.” We produced a single score for each participant by taking the mean of semantic distance across their set of alternative uses.

For the VGT, semantic distance was measured between pairs of noun prompts and verb responses. Semantic distance was computed by calculating the cosine distance between the GloVe embeddings of the noun prompt and verb response. For verb responses given as multiple words (for example, “launching into space” as a response to the noun prompt “swing”), the semantic distance was calculated by taking the mean of the semantic distances between each component word in the response and the noun prompt. Before calculating the semantic distance, verb responses were processed in three steps for consistency: (1) verbs were edited to take the “-ing” form of the verb (for example, “run” was changed to “running”); (2) stop words (e.g. a, at, is, the, which, and on) were removed; (3) for instances where the prompt noun was repeated in the verb response, the repeated noun was removed from the response. For example, using these processing steps, the response of “eat a leaf” to the noun prompt “leaf” was modified to just “eating”). For each participant, we produced a single score by taking the mean of the semantic distance values across the full set of noun–verb response pairs produced by the participant, adapting methods from Prabhakaran et al.^49^. Semantic distance in creative cued VGT has a demonstrated strong relationship to a creativity factor derived from verbal, nonverbal, and achievement-based creativity measures^47^.

### Semantic distance index

Partial Pearson correlations were calculated between the semantic distance measurements of each task (with state and condition as covariates) and showed low to moderate levels of correlations between tasks. To combine semantic distance across all three tasks into a single multifaceted measure of semantic distance, we constructed a composite “Semantic Distance Index” by averaging each participant’s z-scored semantic distance measurements across the three tasks^54,55^.

### Statistical analysis with aligned rank transform

For our omnibus analysis of main effects and interaction, we used the two-way, nonparametric Aligned Rank Transform ANOVA test (ART ANOVA)^61^. Post-hoc pairwise comparison analyses were conducted using nonparametric Aligned Rank Transform multifactor contrast tests (ART-C) corrected for multiple comparisons with Holm-Bonferroni adjustments^62–64^.

### Regression of creative performance on degree of theme incorporation in verbal reports

We ran a multivariate ordinary least squares regression of creative performance on the degree of theme incorporation in verbal reports, along with one continuous control variable (semantic distance) and two binary control variables (sleep and incubation). The regression model was of the form Y = α + (β1 × Degree of Theme Incorporation) + (β2 × Semantic Distance) + (β3 × Sleep) + (β4 × Incubation), where Y was a measure of creative performance (the Creativity Index or one of the individual task ratings), α is a constant, Sleep is a binary variable (1 for sleep participants, 0 for wake participants) and Incubation is a binary variable (1 for incubation group participants, 0 for no-incubation group participants). One participant from the Sleep No-Incubation group was excluded from this analysis because their verbal reports were inaudible during the experiment.

### Assessing verbal report concept incorporation in task responses

We assessed each participant’s verbal reports and task responses to identify instances of direct incorporation of concepts (including concepts beyond the word “tree”) from participants’ mentation during the experimental period in their responses to the creativity tasks. We measure direct incorporation adapting methods from Wamsley et al.^40^.

## Results

### Verbal reports indicate successful incubation of the “tree” theme

During the 45-min experimental period, we collected verbal reports from all participants to record the content of their dreams (sleep groups) or mind-wandering (wake groups) (see Supplementary Table S1 for sample reports). We counted direct references to the “tree” theme in the verbal reports to assess the degree of theme incorporation in their mentation, adapting protocols from Wamsley et al.^40^.

For each participant in the sleep groups, the exact timing of verbal reports and total number of verbal reports collected during the experimental period was determined by the participant’s time needed to transition between wake and N1 sleep and the variable timer triggered after sleep onset. Participants in the SI group (n = 13) provided an average of 5.15 reports, while those in the SN group (n = 12) produced an average of 5.75 (Table 1). All sleep participants had at least 1 report with recalled dream content. In the SI group, an average of 70.3% of each participant’s reports contained a direct reference to the “tree” theme, with an average of 1.25 references to “tree” per report (Table 1). In the SN group, one participant reported a dream including a tree, resulting in an average of 1.4% of reports per participant containing a direct theme reference (Table 1).

We also gathered verbal reports from participants in the two wake state groups. For each participant, the exact timing and total number of verbal reports was determined by the variable timer used in the protocol. Participants in the WI group (n = 12) produced an average of 4.66 reports (with an average of 0.9 references to “tree” per report), while those in the WN group (n = 12) produced an average of 4.00 reports (Table 1). In the WI group, an average of 52.4% of each participant’s reports contained a direct reference to the “tree” theme, while no “tree” reports were obtained from the WN group (Table 1).

### Creative performance differs by state and condition

**Figure 3:**
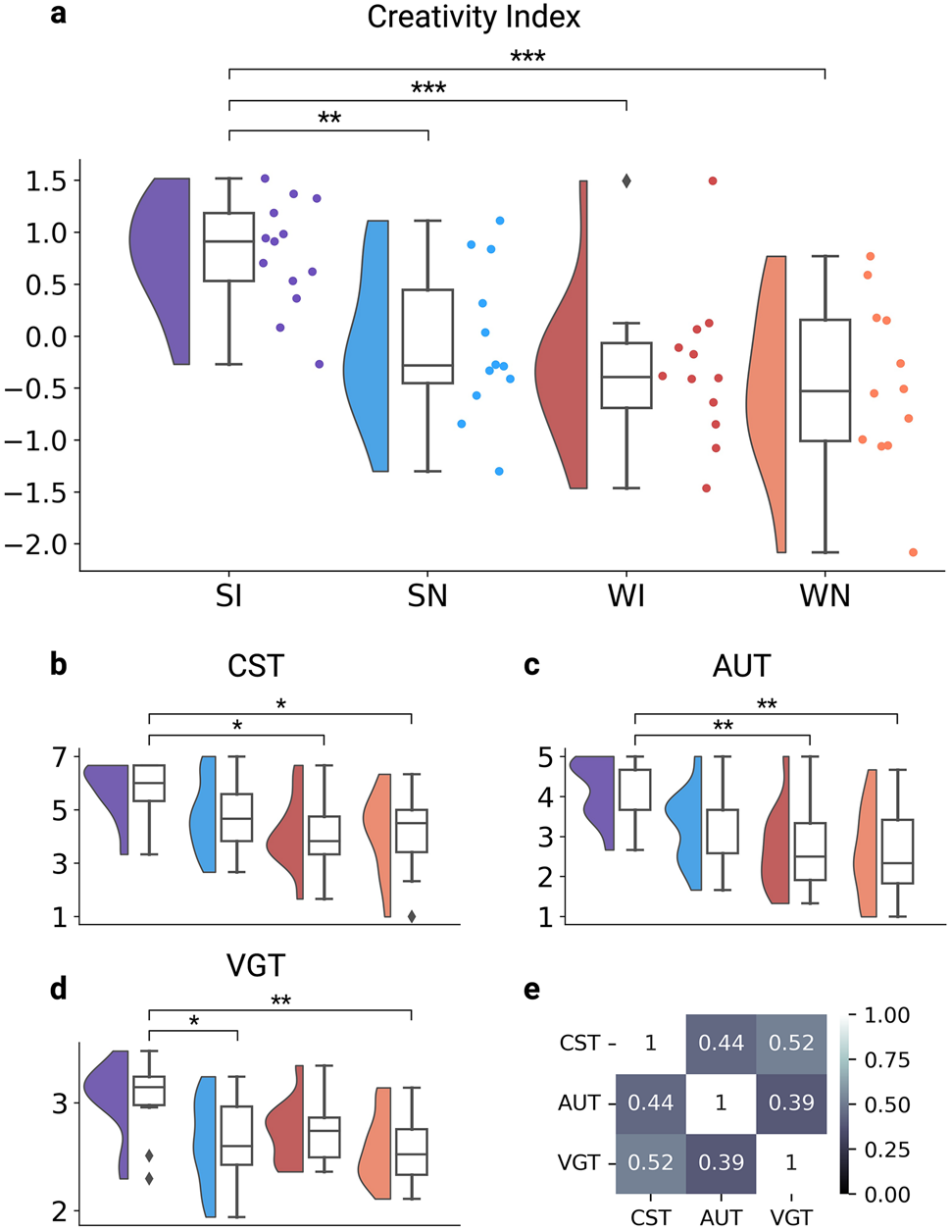
Creative performance metrics. (**a**–**d**) Raincloud plots^65^ comparing creative performance metrics across all groups (SI = Sleep Incubation, SN = Sleep No-Incubation, WI = Wake Incubation, WN = Wake No-Incubation). Diamond markers above and below the box plot indicate outliers. Significance bars generated by an Aligned Rank Transform multifactor contrast test with Holm-Bonferroni correction. (* = p < 0.05, ** = p < 0.01, *** = p < 0.001). (**a**) Creativity Index (weighted mean z-score composite of individual task ratings). (**b**–**d**) Creativity ratings for each of the three tasks. (**b**) CST (Creative Storytelling Task) rated from 1 (low) to 7 (high). (**c**) AUT (Alternative Uses Task) rated from 1 (low) to 5 (high). (**d**) VGT (Verb Generation Task) rated from 1 (low) to 5 (high). (**e**) Partial Pearson's correlation of ratings between the three creativity tasks (controlling for state and condition). All correlation coefficients are significant (p < 0.01).

Following the experimental period, all participants completed the same set of three widely validated creativity tasks (the Creative Storytelling Task, the Alternative Uses Task, and the Verb Generation Task) and their responses were scored by human raters (Table 1, Fig. 3b–d). The moderate positive partial Pearson correlations between task ratings (controlling for state and condition) supported the conclusion that each of the three tasks indexed different aspects of creative performance (Fig. 3e).

To evaluate a multifaceted measure of creativity with broad real-world relevance, we constructed a composite score across the three task ratings for each participant called the Creativity Index (Table 1). A two-way, nonparametric Aligned Rank Transform (ART) ANOVA was performed on the Creativity Index to analyze the effect of state and condition on creative performance (Table 2). There was a significant main effect of both state and condition on creative performance, as well as a significant interaction effect (Table 2). The main effect of state (sleep/wake) showed higher creative performance in the sleep participants than wake participants (p = 0.0002). The main effect of condition (incubation/no-incubation) showed higher performance in the incubation groups than the non-incubated groups (p = 0.015). The interaction between state and condition (p = 0.049) was further investigated with post-hoc comparison tests. An ART multifactor contrast test with Holm-Bonferroni correction revealed that the Sleep Incubation (SI) group exhibited higher creativity than all three other groups: SI-SN (p = 0.009), SI-WI (p = 0.0007), and SI-WN (p = 0.0004) (Fig. 3a, Table 2). None of the other contrasts reached significance.

**Table 2:** Creative performance comparisons.

Omnibus	Creativity Index		CST Creativity		AUT Creativity		VGT Creativity	
	F	p	F	p	F	p	F	p
State	16.39	0.0002***	7.90	0.007**	13.64	0.0006**	5.12	0.029*
Condition	6.33	0.015*	1.14	0.291	3.25	0.078	7.62	0.008**
Interaction	4.08	0.049*	2.21	0.144	2.22	0.144	1.90	0.175

Post-hoc comparisons	t	p	t	p	t	p	t	p
SI–SN	3.23	0.009**	1.94	0.23	2.36	0.09	2.88	0.03*
SI–WI	4.17	0.0007***	3.26	0.01*	3.99	0.001**	2.56	0.06
SI–WN	4.43	0.0004***	2.89	0.03*	4.09	0.001**	3.58	0.005**
SN–WI	0.92	0.74	1.29	0.61	1.60	0.29	− 0.31	0.98
SN–WN	1.18	0.74	0.93	0.72	1.70	0.29	0.69	0.98
WI–WN	0.26	0.80	0.36	0.72	0.10	0.92	1.01	0.96

Statistical results of comparing creative performance metrics. Omnibus: Aligned Rank Transform ANOVA test comparing main effects and interaction of state (sleep/wake) and condition (incubation/no-incubation). Asterisks indicate statistical significance. Post-hoc comparisons: Aligned Rank Transform multifactor contrast tests comparing all pairings of the four participant groups. Asterisks indicate statistical significance after adjusting for multiple comparisons with Holm-Bonferroni corrections (*p < 0.05, **p < 0.01, ***p < 0.001).

To supplement interpretation of the Creativity Index, we also provide the ratings for each of the three tasks (Table 1) along with results from the same statistical testing procedure as used on the Creativity Index (Table 2). The same main effect of state was seen for all three tasks, with sleep participants outperforming wake participants. However, only the VGT showed a main effect of condition (with incubated participants outperforming non-incubated participants) and none of the three tasks showed a significant interaction between state and condition (Table 2). Post-hoc comparisons showed varied results across the three tasks, with significant contrasts occurring where the Sleep Incubation group outperforms another group: SI outperformed WN on all three tasks, WI on the CST and AUT, and SN on the VGT (Fig. 3b–d, Table 2). None of the other contrasts reached significance. Although no interaction effect reached significance on any task, the pattern of post-hoc results suggest that a larger sample size might uncover such interactions.

### Semantic distance differs by state

**Figure 4:**
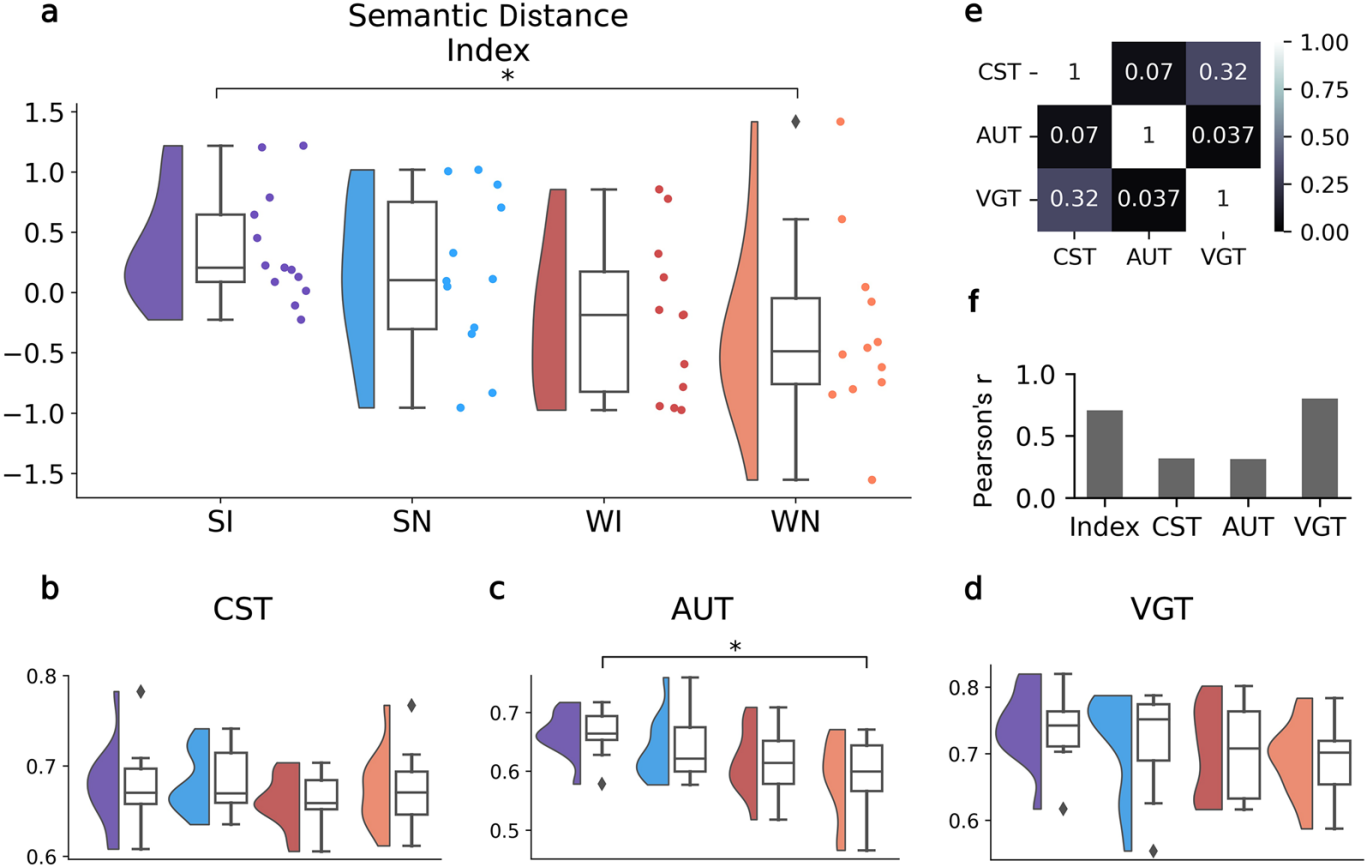
Semantic distance metrics. (**a**–**d**) Raincloud plots^65^ comparing semantic distance metrics across all groups (SI = Sleep Incubation, SN = Sleep No-Incubation, WI = Wake Incubation, WN = Wake No-Incubation). Diamond markers above and below the box plot indicate outliers. Significance bars generated by an Aligned Rank Transform multifactor contrast test with Holm-Bonferroni correction. (*p < 0.05). (**a**) Semantic Distance Index (weighted mean z-score composite of individual task semantic distance measurements). (**b–d**) Semantic distance measurements for each of the three tasks. (**b**) CST (Creative Storytelling Task). (**c**) AUT (Alternative Uses Task). (**d**) VGT (Verb Generation Task). (**e**) Partial Pearson's correlation of semantic distance measurements between the three creativity tasks (controlling for state and condition). Only the CST-VGT correlation is significant (p < 0.05). (**f**) Pearson’s correlation of creativity ratings and semantic distance for the composite index and the three tasks. All correlations are significant (p < 0.05).

We computationally measured the semantic distance between words in task responses (Table 1). The moderate positive partial Pearson correlations between task semantic distance measurements (controlling for state and condition) suggested that participants’ semantic distance was not uniform across the three tasks (Fig. 4e). Therefore, similar to the creativity ratings, we constructed a composite score across the three semantic distance measurements for each participant called the Semantic Distance Index (Table 1). A two-way, nonparametric Aligned Rank Transform (ART) ANOVA was performed on the Semantic Distance Index to analyze the effect of state and condition on semantic distance. There was a significant main effect of state on semantic distance (Table 3). The main effect of state (sleep/wake) showed higher semantic distance in the sleep participants than in the wake participants (p = 0.002). There was not a main effect of condition or an interaction effect. Post-hoc comparisons with an ART multifactor contrast with Holm-Bonferroni correction revealed one significant contrast: SI-WN (0.03) (Table 3, Fig. 4a). None of the other contrasts reached significance.

**Table 3:** Semantic distance comparisons.

Omnibus	Semantic Distance Index		CST Semantic Distance		AUT Semantic Distance		VGT Semantic Distance	
	F	p	F	p	F	p	F	p
State	10.72	0.002**	1.12	0.295	7.89	0.007**	4.260	0.045*
Condition	1.02	0.318	0.21	0.649	3.10	0.085	0.416	0.522
Interaction	0.01	0.941	0.002	0.967	0.47	0.494	0.063	0.802

Post-hoc comparisons	t	p	t	p	t	p	t	p
SI–SN	0.99	0.66	− 0.37	1.00	1.62	0.45	0.40	1.00
SI–WI	2.53	0.07	0.91	1.00	2.36	0.11	1.27	0.84
SI–WN	2.96	0.03*	0.43	1.00	3.21	0.01*	1.95	0.34
SN–WI	1.52	0.41	1.25	1.00	0.73	0.82	0.86	1.00
SN–WN	1.93	0.24	0.78	1.00	1.56	0.45	1.52	0.67
WI–WN	0.42	0.68	− 0.48	1.00	0.84	0.82	0.67	1.00

Statistical results of comparing semantic distance metrics. Omnibus: Aligned Rank Transform ANOVA test comparing main effects and interaction of state (sleep/wake) and condition (incubation/no-incubation). Asterisks indicate statistical significance. Post-hoc comparisons: Aligned Rank Transform multifactor contrast tests comparing all pairings of the four participant groups. Asterisks indicate statistical significance after adjusting for multiple comparisons with Holm-Bonferroni corrections (*p < 0.05, **p < 0.01, ***p < 0.001).

To supplement interpretation of the Semantic Distance Index, we also provide the semantic distance measurements for each of the three tasks (Table 1, Fig. 4) and perform the same statistical testing procedure as we did for the Semantic Distance Index (Table 3). The same main effect of state was seen for the AUT (p = 0.007) and VGT (p = 0.045), wherein sleep participants outperformed wake participants. None of the three tasks showed a significant main effect of condition or a significant interaction between state and condition (Table 3). All post-hoc contrasts failed to reach significance for the CST and VGT (Table 3, Fig. 4b,d). For the AUT, one contrast reached significance in which the Sleep Incubation group outperformed the Wake Incubation group (p = 0.01; Table 3, Fig. 4c).

We also compared the semantic distance measurements with the human-generated creativity ratings by correlating the two composite indices (the Semantic Distance Index and the Creativity Index) and the two metrics for each of the three tasks (Fig. 4f). The correlation between semantic distance and creativity ratings was moderate for the CST (r = 0.21, p < 0.03) and AUT (r = 0.31, p < 0.03) and high for the VGT (r = 0.80, p < 10e−12) and composite indices (r = 0.71, p < 10e−8).

### Degree of theme incorporation in verbal reports as a predictor of creative performance

To evaluate a potential relationship between “tree” dreams and the creative boost observed in the Sleep Incubation group, we analyzed creative performance as a function of the success of the incubation of the “tree” theme. We quantified the degree of theme incorporation for each participant as the average number of direct references to “tree” made per verbal report. To assess the predictive relationship between the degree of theme incorporation and creative performance, we ran a multivariate ordinary least squares regression of creative performance on the degree of theme incorporation (see “Methods”). We ran four regressions with four different creative performance dependent variables (the Creativity Index and the three individual task ratings). We included three key control variables to isolate the specific contribution of the degree of theme incorporation to creative performance. To account for state and condition, sleep and incubation were included as binary control variables. Based on the correlation between semantic distance and creative performance (Fig. 4f), we also included semantic distance as a continuous control variable.

The Creativity Index was well-explained by the model (adjusted R^2^ = 0.60) (Table 4). The three individual tasks demonstrated varying levels of fit with the model (with adjusted R^2^ between 0.27 and 0.72) (Table 4). All of the regressions produced a significant F-statistic (p < 0.001 for all comparisons; Table 4).

**Table 4:** Regression of creative performance on degree of theme incorporation in verbal reports.

	(1) Creativity Index	(2) CST Creativity	(3) AUT Creativity	(4) VGT Creativity
Degree of Theme Incorporation (in verbal reports)	0.260 (< 0.001)*	0.537 (0.004)*	0.423 (0.008)*	0.086 (0.03)*
Semantic distance	0.683 (< 0.001)*	9.862 (0.04*	0.809 (0.72)	4.251 (< 0.001)*
Sleep	0.333 (0.03)*	0.858 (0.01)*	1.011 (0.002)*	0.043 (0.48)
Incubation	0.132 (0.46)	0.04 (0.93)	0.033 (0.92)	0.111 (0.10)
Constant	− 0.388 (0.01)*	− 2.709 (0.42)	1.943 (0.12)	− 0.406 (0.09)
F-statistic	29.57 (< 0.001)*	8.73 (< 0.001)*	7.52 (< 0.001)*	73.89 (< 0.001)*
Adj. R^2^	0.60	0.27	0.30	0.72

Multivariate OLS regressions (with heteroskedasticity robust estimators) of creative performance on the degree of theme incorporation in verbal reports while controlling for semantic distance, state, and condition (*p < 0.05 for indicated regressor coefficient or F-statistic). For the Creativity Index, semantic distance was the Semantic Distance Index. For each of the individual task creativity ratings, semantic distance was the corresponding individual task semantic distance measurement.

For the Creativity Index, we found a significant positive coefficient on the Degree of Theme Incorporation (p < 0.001). This coefficient (β = 0.260) is the average increase in the Creativity Index (mean ± std = 0.0 ± 0.84) for an increase of one in the Degree of Theme Incorporation (while holding semantic distance constant and controlling for state and condition). We also found a significant positive coefficient on Semantic Distance (β = 0.683, p < 0.001), indicating there was, on average, an increase in the Creativity Index with greater semantic distance (holding other variables constant). For our binary control variables, we found a positive significant coefficient on Sleep (β = 0.333, p = 0.03) and a positive but not significant coefficient on Incubation (β = 0.132, p = 0.46) (Table 4). The positive coefficient on Sleep aligns with the significant main effect of state found on the Creativity Index (Table 2). The lack of significance for the Incubation coefficient suggests that the Degree of Theme Incorporation is a better predictor of creative performance than the mere application of incubation.

For the individual task ratings, the regression resulted in varying sets of significant regressors. For all three tasks, we found a significant positive coefficient on the Degree of Theme Incorporation (Table 4). These positive coefficients are the average increases in the creativity ratings for each task for an increase of one in the Degree of Theme Incorporation (holding other variables constant). For the CST and VGT, we also found a significant positive coefficient on the Semantic Distance regressor (Table 4). For the CST and AUT, we found a significant positive coefficient on Sleep (Table 4).

### Incorporation of verbal report content in creativity task responses

To investigate the contribution of mentation during the experimental period to post-sleep creativity task responses, we assessed whether any concepts in each participant’s verbal reports (beyond the word “tree”) was incorporated directly in their task responses, adapting methods from Wamsley et al.^40^. For example, one Sleep Incubation group participant who reported dreaming of “deforestation” and “lumberjacks” later described “deforestation” in their CST response and included “source of income for lumberjacks” in their AUT response (Supplementary Table S3). We report all instances of directly recurring content between the verbal reports and task responses (Supplementary Table S3) and summarize the number of participants with recurring content for each task (Table 1). We found instances of concept recurrence in CST and AUT responses, but none in VGT responses.

In the Sleep Incubation group, we found 8 of 13 participants had verbal report concepts reappear in their CST responses and 4 of 13 participants had concepts reappear in their AUT responses (Table 1). Recurring content included specific types of trees (such as an oak tree), sounds (such as rustling leaves), settings (such as a desert, a specific river, and a kitchen), animals (including cats and turtles), and fantasy elements (such as rapid tree growth or having a body made of wood) (Supplementary Table S3).

We also found recurring content in the CST of 3 of the 12 participants in the Wake Incubation group (Table 1), including specific settings (such as a public park), tree-related actions (climbing trees), and personification of tree parts (such as leaves being compared to eyes) (Supplementary Table S3).

We also found recurring content in 1 of the 12 participants in the Sleep No-Incubation group (on the CST) and 2 of 12 participants in the Wake No-Incubation group (one on the CST and one on the AUT) (Table 1). Recurring content included settings (such as MIT) and objects (such as musical instruments and a black sphere) (Supplementary Table S3).

There was a significant difference in the rate of concept recurrence between all four experimental groups (two-sided Fisher’s exact test, p = 0.024), but no significant differences were found in pairwise post-hoc contrasts with Holm-Bonferroni correction for multiple comparisons (Supplementary Table S4).

## Discussion

We present results that demonstrate that targeted dream incubation (TDI) during N1 sleep can enhance post-sleep creative performance on tasks related to the incubated theme. Our study on the link between N1 dreams and creative performance builds upon a recent scientific study of the creative potential of the N1 sleep stage, in which a period of N1 sleep was found to triple the chance of study participants having a moment of creative insight on a previously studied mathematical task as compared to participants who remained awake^13^. However, a clear link between N1 dream content and the post-N1 creative insight was absent in the study^13^. To investigate the relationship between N1 dream content and post-sleep creative performance, we collected verbal mentation reports from participants during a period of either N1 sleep or wake during which participants were either prompted to think about a specific incubation theme (“tree”) or simply to pay attention to their thoughts. Our 2 × 2 design varying state (sleep/wake) and condition (incubation/no incubation) allowed us to investigate the potential contribution of dream content to the N1 creative sweet spot.

To obtain a multifaceted measure of creative performance with broad real-world relevance, we administered a set of three widely validated creativity tasks, each shown to index a different aspect of creative performance (the Creative Storytelling Task, the Alternative Uses Task, and the Verb Generation Task). Human raters scored participant responses to the three creativity tasks based on task-specific definitions of creativity. We then constructed a composite score across the three tasks called the Creativity Index. We found a main effect of state on the Creativity Index, with sleep participants outperforming wake participants. Broadly, this result corroborates past findings of sleep presenting an optimal environment for creative ideation over time-matched periods of wake^8–11^ and aligns with a recent study specifically identifying N1 sleep as a creative sweet spot^13^. We also found a main effect of condition on creative performance, corroborating past findings that incubation of task-related content confers a creative benefit^66–68^. Post-hoc pairwise group comparisons of the Creativity Index showed that the Sleep Incubation group (who received the N1 targeted dream incubation protocol) significantly outperformed each of the other three groups (Sleep No-Incubation, Wake Incubation, and Wake No-Incubation). Specifically, the Sleep Incubation participants outperforming both the Sleep No-Incubation participants (who slept but did not receive incubation) and the Wake Incubation participants (who received incubation while awake) highlights the benefit conferred by engaging in both N1 and incubation simultaneously.

The next phase of our analysis investigated whether the success of dream incubation was predictive of creative performance. Here, we probed not only whether the application of an incubation protocol confers a benefit, but also to what degree this benefit is dependent on direct incorporation of incubated themes into dreams. This analysis is motivated by past anecdotal reports from thinkers including both Thomas Edison and Salvador Dalí, wherein sleep onset dreams contained explicit images or thoughts that contributed to the creative resolution of complex problems including the design of inventions and works of visual art^4^. We evaluated the predictive relationship between the degree of theme incorporation (quantified as the average number of “tree” references made per dream or wake verbal report) and creative performance. We regressed creative performance on the degree of theme incorporation, while also controlling for state, condition, and semantic distance. We found a positive relationship between sleep and creative performance, corroborating our earlier results where we found a main effect of state on creative performance. We also found a positive relationship between semantic distance and creative performance, corroborating past findings showing greater semantic distance predicts higher creativity ratings^60,69^. Importantly, we found a significant positive relationship between the degree of theme incorporation (the average number of “tree” references made per verbal report) and creative performance, demonstrating that, on average, each additional successful incubation confers an additional creative boost. This evidence—that the degree of theme incorporation positively predicts the level of creative performance—suggests that a dream with theme-related content may itself confer a creative boost on tasks related to that content, in line with past research correlating dreams and creativity^10,15,17,34,35,70^.

Understanding the predictive relationship between the degree of “tree” incorporation and creative performance sheds light on *whether having* such mentation contributes to creative performance, but does not show *how* the specific content in such mentation may contribute to creative performance. For instance, “tree”-related mentation may contribute to creative performance on the CST indirectly by priming a broad associational network of concepts related to trees, or it might instead contribute by providing material for direct incorporation into a creative story about a “tree.” To investigate direct contributions of dream content to task performance, we measured whether specific concepts (objects, settings, or actions beyond simply the word “tree”) appearing in reported mentation reappeared in creativity task responses. We found a significant difference in the rate of concept recurrence between the four experimental groups. We found the highest level of direct incorporation of mentation into creativity tasks in the Sleep-Incubation group, though post-hoc pairwise contrasts with Holm-Bonferroni correction fail to reach significance. These results motivate future research into the degree to which N1 dream content itself can be a source for creative ideation.

Our study also sheds light on a potential mechanism underlying the N1 creative sweet spot. The associative theory of creativity would argue that N1 sleep is an ideal state for creative ideation because it may facilitate heightened associative divergence. Based on this characterization of N1, we hypothesized that there would be greater semantic divergence in task responses from sleep participants than wake participants. We computationally evaluated the semantic distance in task responses and constructed a composite score across all three tasks called the Semantic Distance Index. We found a significant main effect of state on the Semantic Distance Index, with sleep participants showing greater semantic distance in their responses than wake participants. These results support the hypothesis that N1 enables a cognitive state with greater associative divergence, facilitating the exploration of connections between distantly associated concepts. This heightened associative divergence in N1 may be a potential mechanism for promoting creative insight.

Our experiment design presents a number of limitations for interpreting our findings. First, we are limited in our measurement of the *specificity* and *duration* of the benefit of N1 TDI. We use a single prompt (“tree”) for our incubation and our three creativity tasks. While our study suggests that dream incubation confers a creative benefit on tasks related to that theme, the specificity of this creative benefit to a given theme (i.e. “tree”) is yet to be established. It is conceivable that simply asking participants to incubate *any* dream theme would in turn increase attention to their dreams and thus improve post-sleep creativity. Future research including an experimental condition that incubates a dream theme unrelated to the post-sleep creativity tasks could resolve this question. Moreover, future research using themes other than “tree” for both dream incubation and creativity testing is needed to generalize and extend our findings to different subject domains. In addition, our study assessed creativity immediately following the administration of the TDI protocol, limiting our measurement of the duration of the creative benefit. Given that past studies have suggested dream incubation can have effects on dream content and daytime performance up to a week after incubation protocols, future studies should gather data over longer timescales^71,72^. Second, due to a lack of counterbalancing in the administration of our three creativity tasks, our results may be influenced by order effects. Third, due to the small sample size of each group, we are limited in our ability to determine the precise extent of the correlations between creative performance across tasks and between creative performance and semantic distance. Fourth, again due to the small group sizes, we performed nonparametric testing, which reduced our power to detect smaller effects. Future research with larger sample sizes and parametric testing will more powerfully elucidate the effects of sleep and incubation. Fifth, the protocol used for waking up participants in the sleep groups (a variable 1–5 min timer after Dormio-detected sleep-onset N1), along with the lack of EEG in the Dormio device’s physiological recording channels, leaves open the possibility that some awakenings were from N2 instead of N1 sleep. Future research should extend the Dormio system to include N2 detection or apply concurrent EEG recording to more reliably restrict awakenings to N1 sleep.

Finally, there is a fundamental limit to claims of *causality* when experimenting on conscious experience. Standard requirements for a causal scientific claim include a correlation between cause and effect, the effect appearing after its cause, changes in the effect produced by varying the strength of the cause, and a plausible explanation of the process by which this happens^27^. We are interested in understanding the causal effect of dream experience on post sleep cognition, yet when it comes to experimenting on experience, we fundamentally cannot distinguish conscious experience from its underlying neural cause^73,74^. As such, we do not claim to separate dreaming from its underlying neural activation; instead, we claim the neural activation caused by incubating a specific theme, which in turn leads to theme-related dream content, is also causally related to post-sleep performance on theme-related tasks. This is the closest that any scientific study of consciousness can come to claims of causality.

The TDI protocol permits controlled studies with dream content as an independent variable, allowing random group assignment in dream studies. TDI can be performed by any system that has sensors to track sleep stages and a method to deliver audio cues^36^. The Dormio system used in this study offers improvements on older methodologies for capturing N1 mentation, reliably incubating dream themes through the use of well-timed audio cues and further allowing for multiple rounds of hypnagogic dream reporting in a single nap period^36^. It automatically collects verbal dream reports before they are lost to amnesia and avoids the more complete awakening that a written report would require, facilitating an easier return to N1 after each verbal report^36^. Importantly, Dormio has adjustable wakeup parameters so that users do not enter N2 and lose the N1-associated creative boost. We hope the TDI protocol and Dormio device can be used to probe known correlational links between dreaming and post-sleep performance at the causal level.

With our study design and TDI technique, we were able to independently vary dream content without presenting the creativity tasks themselves pre-sleep, where they could act as incubation stimuli. Thus, we have eliminated the possibility that pre-sleep task performance was independently causing participants who performed better post-sleep to dream about the task. This study thus provides the first controlled experimental evidence suggesting a positive contribution of incubating dream content to creative performance.

We hope this study, as well as the TDI protocol that enabled it, inspires a host of experiments probing the contribution of dream incubation to sleep-mediated cognitive processing. In the past, dream experience has too often been explained as the unimportant, and indeed, random result of important nonconscious cognitive processing ongoing in sleep^75–77^. We argue, based on the data reported here, that dreaming—a combination of experiential phenomenology and brain physiology—may contribute causally to the sleep-dependent processes of creativity, learning, and memory, and that it is an important area of study if we want to fully understand these processes.

## Supplementary Information

Supplementary Information.

## Data Availability

The datasets generated in the present study are available from the corresponding author on reasonable request.
