# Supplementary material for: Targeted dream incubation at sleep onset increases post-sleep creative performance
Source: Sci Rep. 2023 May 15;13:7319. doi: 10.1038/s41598-023-31361-w (PMC10185495; doi:10.1038/s41598-023-31361-w)
Supplement: Supplementary file 1 — Supplementary Information. [file 41598_2023_31361_MOESM1_ESM.pdf]

Supplementary Materials

Targeted dream incubation at sleep onset increases post-sleep creative performance

Adam Haar Horowitz*[,a,b,c], Kathleen Esfahany*[a,d], Tomás Vega Gálvez[a], Pattie Maes[a] & Robert Stickgold[b,c]†

[a] MIT Media Lab, Massachusetts Institute of Technology, Cambridge, MA, 02139, USA
[b] Center for Sleep and Cognition and Department of Psychiatry, Beth Israel Deaconess Medical Center, Boston, MA, 02215, USA
[c] Department of Psychiatry, Harvard Medical School, Boston, MA, 02115, USA
[d] Department of Brain and Cognitive Sciences, Massachusetts Institute of Technology, Cambridge, MA, 02139, USA

†Corresponding Author: rstickgold@hms.harvard.edu

* denotes equal contribution

Experimental Procedures

1.1 Experimental Instructions Read to Subjects

Below are the specific instructions given to the subjects in each experimental group. As the instructions are important for effectively initiating dream incubation and hypnagogic imagery, attempts at replication should closely mirror the instructions below:

Group 1 (Sleep Incubation): "This experiment is investigating the relationship between mental rest and cognitive flexibility. Varied imagery, memories, words, or bodily feelings may come up throughout the experiment. The aim of this exercise is to observe them, stay with them, follow them lightly and see where they go. One thing not to worry about is questioning whether you are asleep. This period in between sleep and wake feels to some people like sleep, to others just like relaxation or mind wandering. All are completely fine, just watch your mind and relax. Sleep cannot be forced, just allowed. Head towards sleep, but don't worry at all where you are in it, just relax."

"After you lie down, you will be asked to think of a theme. Relax, hold that theme in your mind. A few times, you will be told you are falling asleep and reminded of the dream theme. These prompts are not to wake you up fully, just to make sure you do not descend into deep sleep, and to keep you aware so that you can keep observing your mind. Just stay still when the prompts come. Again, we're interested in your thinking in this semi-lucid period. Whenever you are prompted to report, please just vocally report what was going through your mind, and report whether you think you were asleep, by either saying awake, halfway or asleep. Then relax and drift towards sleep again."

Group 2 (Sleep No-Incubation): "This experiment is investigating the relationship between mental rest and cognitive flexibility. Varied imagery, memories, words, or bodily feelings may come up throughout the experiment. The aim of this exercise is to observe them, stay with them, follow them lightly and see where they go. One thing not to worry about is questioning

whether you are asleep. This period in between sleep and wake feels to some people like sleep, to others just like relaxation or mind wandering. All are completely fine, just watch your mind and relax. Sleep cannot be forced, just allowed. Head towards sleep, but don't worry at all where you are in it, just relax."

"After you lie down, you will be asked to observe your thoughts. Relax, and see where your thoughts go. A few times, you will be told you are falling asleep and reminded to observe your thoughts. These prompts are not to wake you up fully, just to make sure you do not descend into deep sleep, and to keep you aware so that you can keep observing your mind. Just stay still when the prompts come. Again, we're interested in your thinking in this semi-lucid period. Whenever you are prompted to report, please just vocally report what was going through your mind, and report whether you think you were asleep, by either saying awake, halfway or asleep. Then relax and drift towards sleep again."

Group 3 (Wake Incubation): "This experiment is investigating the relationship between mental rest and cognitive flexibility. Varied imagery, memories, words, or bodily feelings may come up throughout the experiment. The aim of this exercise is to observe them, stay with them, follow them lightly and see where they go. One thing not to worry about is questioning whether you are mind wandering or focused. All are completely fine, just watch your mind and relax."

"After you close your eyes, you will be asked to think of a theme. Relax, hold that theme in your mind. A few times, you will be told to observe your thoughts and reminded of the theme. These prompts are just to keep you aware so that you can keep observing your mind. Just stay still when the prompts come. Again, we're interested in your thinking in this period of mental rest. Whenever you are prompted to report, please just vocally report what was going through your mind. Then relax and let your mind drift again."

Group 4 (Wake No-Incubation): "This experiment is investigating the relationship between mental rest and cognitive flexibility. Varied imagery, memories, words, or bodily feelings may come up throughout the experiment. The aim of this exercise is to observe them, stay with them, follow them lightly and see where they go. One thing not to worry about is questioning whether you are mind wandering or focused. All are completely fine, just watch your mind and relax."

"After you close your eyes, you will be asked to observe your thoughts. These prompts are just to keep you aware so that you can keep observing your mind. Just stay still when the prompts come. Again, we're interested in your thinking in this period of mental rest. Whenever you are prompted to report, please just vocally report what was going through your mind. Then relax and let your mind drift again."

1.2 Prompts for Dream Incubation and Reports

Group 1: Sleep Tree-Incubation involved a prompted hypnagogic nap, wherein we used the Dormio system to incubate the dream theme "tree." Upon lying down, the Dormio web app instructed these participants to "*Think of a Tree.*" Once N1 sleep was detected by the system, a variable timer was triggered. This timer instigated wakeups from 1:00 to 5:00 minutes after N1 detection. At the end of this time, the computer audio alerted participants they were falling

asleep ("*You're falling asleep*"), asked them to verbally report the thoughts they were currently having ("*Please tell me, what's going through your mind*"), and recorded their response. Once participants finished speaking, the system asked about their sleep state ("*And were you asleep?*"), to which participants had been instructed to respond with 'Awake', 'Halfway' or 'Asleep'. The system then instructed them to think of the dream prompt ("*Remember to think of a tree"*) and to go back to sleep ("*You can fall back asleep now*"). This loop of events was repeated for 45 minutes, enabling multiple entries into and exits from hypnagogia. At the end of the last loop, the experimenter instructed the participant to wake up fully.

Group 2: Sleep No-Incubation involved a hypnagogic nap without any suggested theme, wherein we used the Dormio system solely to extend the hypnagogic period across serial sleep onsets and to capture dream reports. The Dormio system functioned as it did in Condition 1, except the prompt, "Remember to think of a Tree" was replaced with "Remember to observe your thoughts."

Group 3: Wake Tree-Incubation involved a prompted period of time-matched wake. Participants sat upright with head unsupported (so the experimenter could survey for muscle tone loss, which would indicate sleep onset), eyes closed, and were instructed to stay awake. Participants were instructed "not to worry about controlling your mind, mind wandering is completely fine, just watch your mind and relax." The Dormio web app then instructed these participants to "think of a Tree". Once 7:00 minutes had passed, approximating sleep onset time, a variable timer was triggered from 1:00 to 5:00 minutes. At the end of this timer window, the computer audio alerted participants and asked them to verbally report the thoughts they were currently having ("Please tell me what's going through your mind"), and their response recorded. Once participants finished speaking, the system prompted them again, "Remember to think of a tree."

Group 4: Wake No-Incubation involved a period of time-matched wake without any suggested theme. Participants sat upright with eyes closed. The Dormio system functioned as it did in Condition 3, except replacing the prompt, "Remember to think of a Tree," with "Remember to observe your thoughts."

Dormio Device

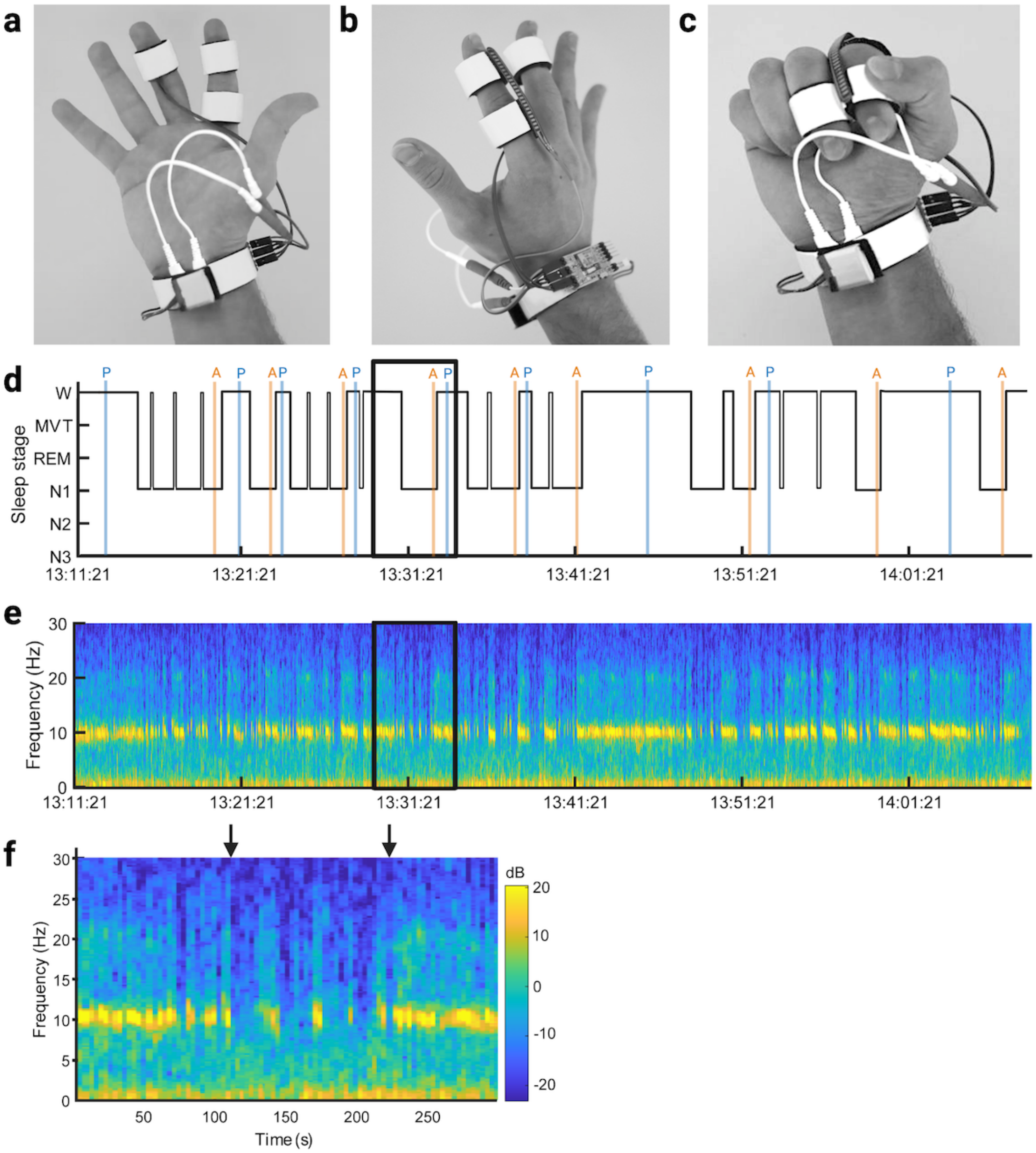

Figure S1. Dormio device and polysomnography recording. a-c. Dormio device. Sensors in handworn Dormio device design facilitate measurement of EDA, HR, and muscle flexion. The minimally constraining design enables more comfortable sleeping than devices worn on the head. Images by Oscar Rosello. **d.** Sample hypnogram displaying sleep staging throughout the experimental period showing the participant repeatedly transitioning between wakefulness and N1 sleep. Sleep staging based on concurrent polysomnography EEG recording. Blue "P" bars mark the Dormio system prompt delivered prior to each incubation period after collection of a verbal report from the participant. Orange "A" bars mark the Dormio-initiated awakening followed by a request for a dream report. **e, f.** EEG recording during a Dormio incubation session. **e.** Multitaper spectrogram of the frequency dynamics of the EEG signal during the incubation period. Spectrogram derived from activity at electrode O1 (left occipital

lobe). Note the attenuation of alpha (~10Hz) rhythm activity during periods of N1 sleep. **f.** Enlarged view of spectral dynamics during a 5 minute incubation period (indicated with a black box in panel e). The incubation period began while the participant was awake. The participant then entered N1 sleep (first arrow), characterized by the marked reduction in the alpha rhythm. A few minutes later (second arrow), the Dormio woke the participant and requested a dream report, which it recorded, and the alpha rhythm reappeared.

Phenomenological Reports

Table S1. Verbal Reports. Examples of verbal reports following report prompts given by the Dormio device.

Participant	Group	Verbal Reports
10	Sleep Incubation	Awakening 1: Trees, many different kinds, pines, oaks<br>Awakening 2: Who I'm going to have over for dinner on Saturday, and occasionally trees, and how I'm not falling asleep<br>Awakening 3: A tree from my childhood, from my backyard. It never asked for anything.<br>Awakening 4: Trees splitting into infinite pieces<br>Awakening 5: I'm in the desert, there is a shaman, sitting under the tree with me, he tells me to go to South America, and then the tree…
37	Sleep No-Incubation	Awakening 1: The desert<br>Awakening 2: I was thinking about how someone else would have to fall asleep here<br>Awakening 3: The sea, sharks, lots of movement<br>Awakening 4: Clouds, and movement and mhmm<br>Awakening 5: The mothering feeling… in the middle… safe<br>Awakening 6: I lost it! I wasn't thinking of anything.
19	Wake Incubation	Report 1: I'm thinking about an X Files episode where a creature ties people to trees<br>Report 2: Thinking about climbing a tree and reading at the top of it<br>Report 3: I'm thinking about walking through orange and almond groves<br>Report 4: The adventure I'm going to have this weekend<br>Report 5: I'm thinking about the tree that the peach grew on in James and the Giant Peach
42	Wake No-Incubation	Report 1: Oh I'm just awake<br>Report 2: Thinking about what I have to do tonight<br>Report 3: I was thinking about the election<br>Report 4: I was thinking about the itch on my ear<br>Report 5: I was thinking about thinking about what I should be thinking about

Table S2. Post Experiment Reports. Examples of reports given after the conclusion of creative tasks regarding dream (sleep groups) or mind-wandering (wake groups) experiences.

Participant	Group	Post-Experiment Report
11	Sleep Incubation	"My dream was pleasant and mysterious. I never knew what the next part of my dream was going to be. My dream did involve a tree. I was following the roots with someone and the roots were transporting me to different locations. At each location I was trying to find a switch. It was unclear why I had to turn on the switch, but at the final location a window with a bright light was revealed. I saw a familiar face, but I couldn't place where I'd seen them. In the background, the moon was shining bright and illuminating the face. I was dreaming this while awake and not fully asleep...I could hear myself talking to someone about finding a switch. I could hear my breathing, my footsteps, the wind, and an air conditioner. When I bumped into objects, I can hear the noise of the collisions. I could hear the roots of the tree pulsating with energy as if they were leading me to some location."
3	Sleep Incubation	"I think it's really, really useful for creativity...at the beginning of my dreaming experience I was seeing scenes that were the same size and functionality of real trees, but then the second time I was much bigger than the trees and I could eat them like finger food. You wouldn't come up with that idea at the beginning, but this time I had hundreds of them. The hope is to break out of the banal stories. Which is why I liked this experiment. I'm afraid when I walk out that I will see everything changing like fantasies. But yeah why not. Who says that I must live in the world that everyone is living in? I could write it as if I'm living in a novel."
6	Sleep Incubation	"I particularly remember feeling that my consciousness was almost entirely untethered when I thought about the subject of my post-sleep story about a tree - when a person began exploring the freedom of space through tree imitation, before collapsing in a twisted heap and fully becoming a tree. Then, they proceeded to explore four dimensions - which was thoroughly confusing but a lot of uninhibited fun."
12	Sleep Incubation	"I'd start with a tree, I'm like thinking of a tree, for the first few minutes I didn't really go anywhere with the tree, I was just looking at it, it was like really colorful, but each time it woke me up, the path and depth in terms of how much it became a story went deeper. I started to go down a story path every time the word tree was mentioned, and when I was told that I was sleeping and to think of a tree again I switched back to the tree and took a different path. Very interesting, relaxing, and really made me think...I felt much more creative than usual. I never really think of myself as a creative person but it felt easier to think of abstract things and stories, like it just came to me."
37	Sleep No-Incubation	"I feel like my story was far more open and interesting than it would have been regularly, and that the thoughts I had flowed into each other much more easily than what otherwise might have been."
28	Sleep No-Incubation	"I think it would be good to think about ideas for art in this state because everything feels looser and your mind goes places it might not go otherwise. Even reflecting on your day feels good because the thoughts don't seem permanent...my thoughts kept jumping around so I didn't really have a normal sense of time. I don't think I really had any sense of time. It felt shorter than 45 min when the experiment ended...I let myself

		think about anything I wanted to and my mind felt very relaxed because I was sort of asleep. It definitely felt different from my normal cognitive state. I think I could see my whole self at some points, so kind of like an out of body experience.”
29	Sleep No-Incubation	“I feel more at ease. And as compared to earlier today - when I couldn't even read more than a few pages without feeling distracted - I feel calm. I feel creative. The writing activity especially was very calming, and makes me feel like that sort of random thought exercise can be a source of inspiration for me moving forward. I just feel more in tune with my experiences and memories - and feel like I want to be more artful... I definitely didn't feel like I had been asleep for 45 minutes. I felt it was more likely around 10-15 minutes. So in that sense, dreams felt a lot slower to me. I didn't really imagine my body. At some points it felt like I wasn't even there, and I was just a pair of eyes observing the memories and world around me - without necessarily physically being there. It's like I was just a visitor, looking at my life as if it were in an enclosure. Life is very beautiful, but sometimes when we get lost in the minutiae of daily activities - we forget that. I felt like I was seeing a highlight reel of peculiar memories, and it really made me want to be more reflective about my life and really decide what I do on an everyday basis with intention and purpose. I just felt much more relaxed during the experience. I didn't feel inhibited by any of the constraints I feel when I have to convey experiences with actual words - it felt like I could communicate without any sort of language (verbal or otherwise) - I just understood everything around me for what it was.”
33	Sleep No-Incubation	“I went from imagining something that I thought might induce a dream, to beginning to dream about that imagined thing. Then, there would be a transition from imagined experience to dream, sometimes that was continuous, and sometimes not continuous at all. When not continuous, my mind would jump from what I had been imagining to something entirely different, and this lack of control indicated to me that I was dreaming. I never lost full awareness of where I was, such that when I was woken I was not surprised to find myself in this reality...However, I seemed to have some kind of creative inspiration while I wrote that seemed somewhat self directed similar to how dreams are. Certainly, I thought of some strange things that I would never have thought about were I not somewhat asleep. I had a dream that I was hovering through the air, and throwing what were like miniature bombs to the ground that would explode into literal mushrooms, not mushroom clouds. I consider ideas like these to be creative.”
35	Sleep No-Incubation	“I had a lot of strong images and some narrative stuff- didn't experience any big hypnic jerk but I guess some little ones. My mind wandered a fair amount from physical locations that were made up to ones that resembled ones I knew. I remember a rather horrifying story from the experience of a train with a pig's face and a piglet who was talking to me and saying I didn't understand what it was like for my mother to be taken away. There was a period of time in which there was narration that sounded a lot like an Oliver Sacks book and like his voice and I had some v mild hallucination type things of colors fluctuating in a thermal-imagery type way. I daydreamed about things I wanted to do in the coming weeks. I saw myself turn around, wearing a veil. I saw maxwelton and bone caves with swing sets at the entrances, and my friends who I just went on a caving trip with sitting in them. I imagined several strange and implausible things and honestly can't remember 90% of them just bc the images went by so quickly”

19	Wake Incubation	“I had a mostly pleasant experience. During the first portion, I found my mind mostly wandering between the task at hand (thinking about trees) and thinking about the study overall and thinking about my heartbeat/breathing...I thought about the word t-r-e-e and I would recall past experiences when I was around trees. It was interesting how I often think of trees as secondary items that are in the environment around me but not really primary. I can't say the thoughts I had were creative.”
42	Wake No-Incubation	“My thoughts were about recent events including the experiment itself. I thought about what I should think about and tried to guess what would trigger the device. I also felt a sense of tightness in my stomach and wondered if it's because I was overly concentrated on my thoughts. I wasn't really trying to be creative. I felt more of what my body is feeling than if I had my eyes open.”

Recurring concepts between verbal reports and task responses

Table S3. Verbal reports and task responses with recurring concepts.

Participant	Group	Selected Verbal Reports	Relevant Quotes		Key recurring themes, concepts, or settings
			CST	AUT	
1	Sleep Incubation	**Report 2 of 8:** "I'm walking through a forest"<br><br>**Report 6 of 8:** "I am in a huge field of tree stumps… I walked through a gray building which was cutting the trees down.. I was looking for people but it was just a machine."<br><br>**Report 8 of 8:** "I am seeing lumberjacks. Will they have jobs now that there is the deforestation machine?"	"Two hikers are walking through the Amazon… They stumble upon a large, almost plain-like landscape where the entire area had been deforested… They walk through and realize there are no humans there but only machines that are chopping trees, making them into paper, and shipping them out, all on their own…"	"Source of income for lumberjacks"	Deforestation, deforestation machine, lumberjacks
2	Sleep Incubation	**Report 1 of 3:** "Cats were going on trees and not coming back"<br><br>**Report 2 of 3:** Report "People are burning down trees"	"Girls used to come to these trees with their cats and cats used to climb up the trees and refuse to come back down."<br><br>"Then came the time of more human expansion, when people started burning down the bulk of trees."	"Burning"	Cats in trees, burning trees
3	Sleep Incubation	**Report 2 of 5:** "There's rustling and I'm under the tree and there's leaves"<br><br>**Report 4 of 5:** "I'm trying to turn something off. I'm in the kitchen with people and friends and I can see the tree roots coming in through the ceiling, the walls, the floor"	"They were sitting under the tree listening to the leaves rustle… they were suddenly transported underground where the tree's roots expanded almost infinitely in every direction. The tree instructed them to follow a path which led them to an abandoned kitchen that seemed to pause in time… the purple walls of the kitchen were covered in the roots. The roots wound in all directions on the walls. Roots were dangling from the ceiling like chandeliers. After taking in the surroundings they began to search for a switch. they felt prompted to flip the switch… They were transported back to the tree. This time they sat in the branches amongst the trees. They continued to listen to the leaves rustling, reflecting on		Rustling leaves, tree roots, tree roots expanding in a kitchen

			their trip to the tree's roots…”		
4	Sleep Incubation	**Report 4 of 5:** “I was lying on the grass with my face down and then I somehow left my body and then was painting the trunk of a tree.”<br><br>**Report 5 of 5:** “I was inside a trunk as if the trunk was a beautiful material'	“There was a kid playing in his house's garden… on the grass next to the one large tree in the garden, while lying down, face, belly and cheek against the grass, feeling the enormity of the tree rising up from the ground...”	“a vertical coffin (its trunk)”	Lying face down on the grass, being inside a tree trunk
5	Sleep Incubation	**Report 1 of 5:** “I can see an oak tree”<br><br>**Report 3 of 5:** “That my body is made of wood and older and scarred”	“There is an oak king. He wears a crown of leaves… at times his body is made of wood and at times it is made of light…”	“Body”	Oak tree, body made of wood
8	Sleep Incubation	**Report 2 of 3:** “Turtles”	“The turtles love the beach, but it is dangerous for them to be on the shore because of predatory birds, so they hide in the trees when they are around...”		Turtles
10	Sleep Incubation	**Report 5 of 5:** “I'm in the desert there is a shaman sitting under the tree”	“Out of the desert and into the sun, a seed was born, and found into the unknown…”		Desert setting
13	Sleep Incubation	**Report 2 of 7:** “Riverside imagery, lots of random flashbacks”	“Walking by the water and there are trees along it. It's the Charles River near MIT…”		Riverside setting
19	Wake Incubation	**Report 2 of 5:** “Thinking about climbing a tree and reading at the top of it”	“A group of adventurers are walking in a forest, onto their next mission… suddenly, they feel a presence surrounding them… wolves. They are being hunted by a pack of wolves… the adventurers are outnumbered… Feeling like they are trapped they have to think quickly. Climb! One of the adventurers yells out. They start climbing the trees as fast as possible. They reach a safe distance at the top of the trees…”		Climbing on trees
23	Wake Incubation	**Report 4 of 5:** “My mind keeps circling back to a weeping willow, both the children's book and the one in the public garden in Boston”	“After work every Tuesday and Thursday, when we did not have band practice, Tyler would go to the Boston Common. He would sit under a big birch tree on top of a modestly sloping hill to smoke out of his favorite vape pen…”		Boston common setting

26	Wake Incubation	**Report 5 of 5:** "Thinking about how each leaf is a photoreceptor, like each leaf is an eye like they have thousands of eyes"	"A thousand eyes looking at the sun shining through intricate supportive lashes. Humanoids like to pick the leaves of the trees and squish them in their hands - like squishing eyeballs of a giant…"		Leaves as eyes
36	Sleep No-Incubation	**Report 6 of 8:** "Speaking to my wife and doing this meditation I do where I see a black sphere"	"The tree lived in a deep, dark, black hole. No, not a hole...a sphere. The place where past and future intersect, where long limbed creatures move like spiders, crawling through the darkness…"		Black sphere
47	Wake No-Incubation	**Report 2 of 5:** "I was playing guitar, hearing music, thinking about flying in the sky. I feel relaxed."		"Instrument"	Musical instrument
49	Wake No-Incubation	**Report 3 of 4:** "Walking through the courtyard at MIT"	"I saw this huge tree around MIT, near the Hayden library. It stands as a gigantic structure and it never loses a leaf…"		MIT setting

Table S4. Group comparison of concept recurrence rates with pairwise Fisher's exact test (with Bonferroni-Holm adjustment).

Pairwise Comparison	p	p (adjusted)
SI - SN	0.011	0.067
SI - WI	0.111	0.222
SI - WN	0.041	0.124
SN - WI	0.59	0.885
SN - WN	1	1
WI - WN	1	1
